# Harnessing the liver to induce antigen-specific immune tolerance

**DOI:** 10.1007/s00281-022-00942-8

**Published:** 2022-05-05

**Authors:** Cornelia Gottwick, Antonella Carambia, Johannes Herkel

**Affiliations:** First Department of Medicine, University Medical Centre Hamburg-Eppendorf, Hamburg, Germany

**Keywords:** Autoimmune disease, Immunotherapy, Immune tolerance, Scavenger cells, Nanomedicine, Antigen presentation

## Abstract

Autoimmune diseases develop when the adaptive immune system attacks the body’s own antigens leading to tissue damage. At least 80 different conditions are believed to have an autoimmune aetiology, including rheumatoid arthritis, type I diabetes, multiple sclerosis or systemic lupus erythematosus. Collectively, autoimmune diseases are a leading cause of severe health impairment along with substantial socioeconomic costs. Current treatments are mostly symptomatic and non-specific, and it is typically not possible to cure these diseases. Thus, the development of more causative treatments that suppress only the pathogenic immune responses, but spare general immunity is of great biomedical interest. The liver offers considerable potential for development of such antigen-specific immunotherapies, as it has a distinct physiological capacity to induce immune tolerance. Indeed, the liver has been shown to specifically suppress autoimmune responses to organ allografts co-transplanted with the liver or to autoantigens that were transferred to the liver. Liver tolerance is established by a unique microenvironment that facilitates interactions between liver-resident antigen-presenting cells and lymphocytes passing by in the low blood flow within the hepatic sinusoids. Here, we summarise current concepts and mechanisms of liver immune tolerance, and review present approaches to harness liver tolerance for antigen-specific immunotherapy.

## Autoimmune diseases and antigen-specific immunotherapy

Autoimmune diseases are caused by a cell- or tissue-damaging immune response of T or B lymphocytes recognising self-antigens [1]. These conditions can affect virtually any tissue of the body, causing a large variability of symptoms and injuries. Accordingly, more than 80 different autoimmune diseases can be distinguished depending on the target structures of the autoimmune attack [2]. Most of these conditions are rare diseases, but there are also more common diseases, such as rheumatoid arthritis, type 1 diabetes, multiple sclerosis or systemic lupus erythematosus. As a group, autoimmune diseases affect about 5–10% of the populations in Western countries [1, 2]. For unknown reasons, the prevalence of autoimmune diseases seems to increase [3]. While some autoimmune diseases can be fatal, virtually all of them impose major health impairments on affected individuals, commonly requiring lifelong medical care. As a result, these diseases also impose a heavy financial and emotional burden on patients and their families, and hence also contribute significantly to the costs incurred by healthcare systems.

**Fig. 1:**
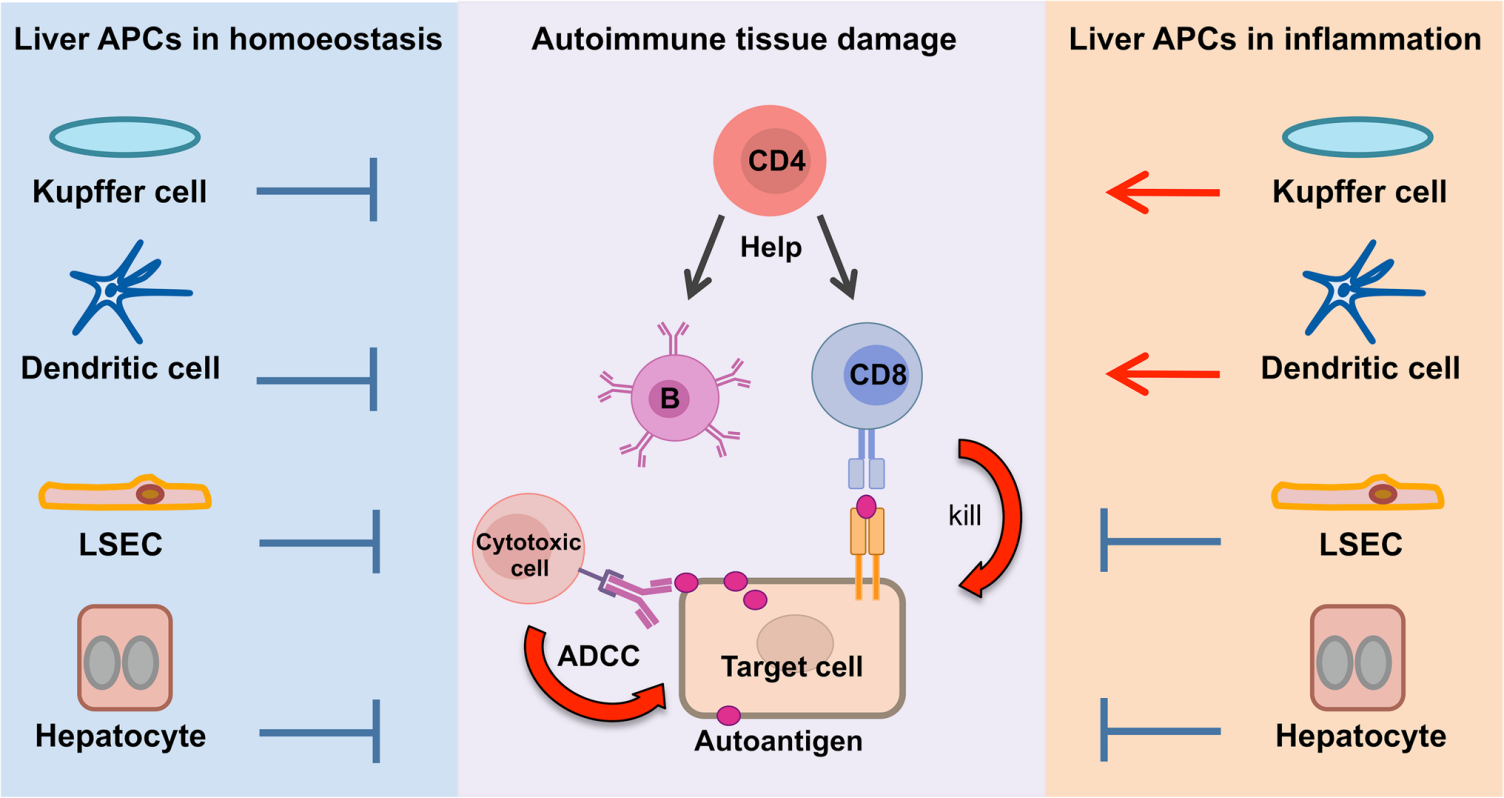
Liver antigen-presenting cells as regulators of autoimmunity. Autoimmune diseases are caused by an adaptive immune response to autoantigens producing damage of target cells or organs. Autoantibodies produced by autoreactive B cells can cause tissue damage indirectly by binding to self-antigens and subsequently activating cytotoxic effector cells by antibody-dependent cell-mediated cytotoxicity (ADCC). Alternatively, cell damage is directly caused by autoreactive CD8 T cells releasing cytotoxic activities upon recognition of self-peptides on MHC I molecules of the target cell. In some cases, CD4 T cells can also become cytotoxic, but they are more relevant for providing help to autoreactive B cells and CD8 T cells upon recognition of self-peptides on MHC II molecules. Typically, CD4 T cell help is required for the development of autoimmune diseases and to maintain damaging autoimmune responses. In homoeostatic conditions, liver antigen-presenting cells, including Kupffer cells, dendritic cells and liver sinusoidal endothelial cells (LSECs), which take up and present autoantigen peptides on MHC molecules to autoreactive T cells, but also hepatocytes can induce T cell tolerance and suppress inflammatory activities, offering opportunities for therapy. However, under inflammatory conditions and liver injury, Kupffer cells and dendritic cells become critical drivers of pathogenic lymphocyte activation and inflammation. In contrast, tolerance-induction by LSECs and hepatocytes is considerably more robust, and it was shown that LSECs remain tolerogenic unless virally infected

A common feature of all autoimmune diseases is the recognition of self-antigens by T or B cells leading to cell damage (Fig. 1). However, autoreactive T and B cells can also be found in healthy subjects, often at similar frequencies as those found in patients with autoimmune disease [4–6]. Of note, autoreactive T cells from healthy animals have been demonstrated to cause autoimmune disease upon activation [7, 8]. Thus, autoreactive lymphocytes are part of the mature lymphocyte repertoire in healthy individuals, and autoimmune inflammation seems to be kept at bay physiologically by regulating the activity of autoreactive lymphocytes [9]. This is mainly achieved either by restricting autoantigen presentation to levels that keep autoreactive T cells ignorant of their cognate antigens [10], or by specialised autoreactive T cells, so-called regulatory T cells, that can suppress conventional autoreactive T cells [11, 12]. Regulatory T cells can be generated in the thymus, but also in the periphery [13], and notably in the liver [14]. It is therefore assumed that autoimmune diseases develop from dysfunctional immune regulation [15]. As a consequence, novel approaches to the therapy of autoimmune diseases aim at restoring immune regulation [16].

Traditional therapies for autoimmune diseases have relied on non-specific immunosuppression that broadly reduces the immune response, placing patients at increased risk of infection or cancer, notably after the required long-term treatment [16]. Alternatively, immunomodulatory biologicals are given that often produce high costs, which can amount up to several tens of thousands of Euro per year. These treatments usually need to be given continuously and lifelong; nonetheless, they do not cure the disease, but rather only retard disease progression. Thus, there is a high need for improved and ideally causative therapies, which might best be matched by antigen-specific immunotherapies that selectively suppress only the pathogenic immune reactions, leaving general immunity unaffected [17].

There has been considerable effort to develop antigen-specific immunotherapies, as summarised in [17, 18], and several approaches are currently being tested in clinical trials. To induce antigen-specific tolerance, most of these approaches make use of physiological antigen presentation in order to promote tolerance [18]. However, autoimmune disease patients are not in physiological state, but in a state of inflammation and impaired tissue function [19], which can impair immune regulation and the tolerance-inducing function of antigen-presenting cells [20, 21]. Thus, there is a possible risk that these tolerogenic treatments are less effective, when used in a state of inflammation, or even might induce disease exacerbation. Therefore, it is of critical importance to utilise only robust tolerance mechanisms that rely on antigen-presenting cells with low plasticity, notably under inflammatory or stress-associated conditions. In our opinion, one of the best ways to achieve that is to utilise liver tolerance, as explained below.

## Liver tolerance

**Fig. 2:**
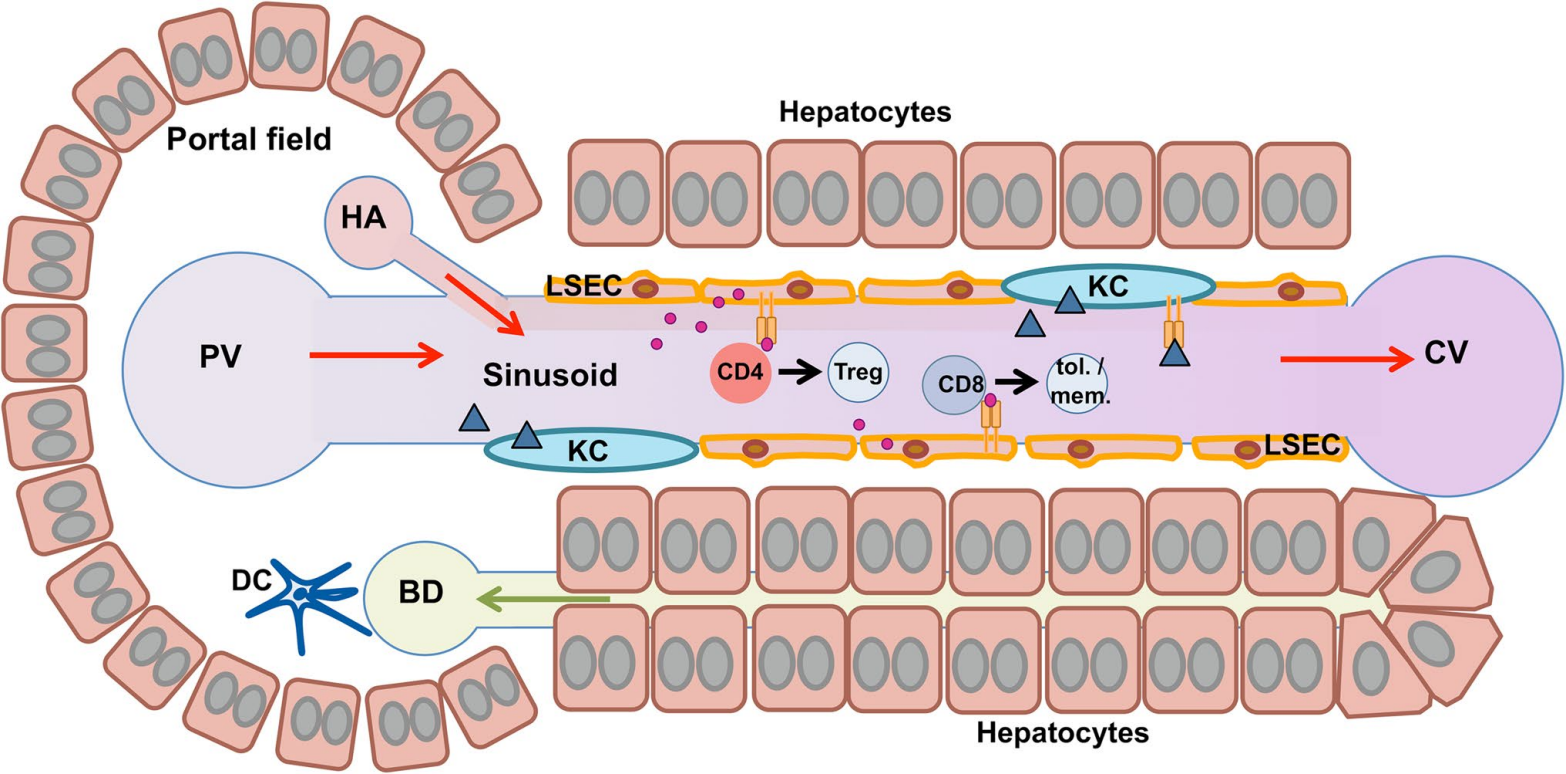
Hepatic antigen-presenting cells in anatomical context. Blood flow (red arrows) enters the liver sinusoids through the portal vein (PV) and the hepatic artery (HA) and leaves through the central vein (CV). The hepatic sinusoids are lined by the liver sinusoidal endothelial cells (LSECs), which are scavenger cells clearing the blood from small particles and macromolecules by receptor-mediated endocytosis. LSECs present collected antigens to lymphocytes, producing a state of immune tolerance. CD4 effector T cells (CD4) can be transformed into regulatory T cells (Treg) through TGF-beta signals. CD8 T cells (CD8) can become tolerant or memory T cells (tol./mem.). Kupffer cells (KCs) reside in the lumen of the hepatic sinusoids and facilitate the removal of larger blood-borne particles by phagocytosis. They also present collected antigens to lymphocytes, producing tolerance. Dendritic cells (DCs) predominantly locate in the portal fields, and often close to bile ducts (BD), where they function as sentinels guarding the integrity of the biliary epithelium. DCs are antigen-presenting cells, producing tolerance in homoeostatic conditions, but readily promote inflammation upon sensing of cell damage or infection. As LSECs and KCs are the predominant cells in sinusoidal blood, it is easier to target those than liver DCs with vectors or carriers for antigen-specific immunotherapy

The liver is the central metabolic organ positioned to directly receive gut-derived blood through the portal vein, carrying nutrients and dietary components, but also microbes and microbial products (Fig. 2) [22]. It contains the largest collection of scavenger cells in the body, consisting mainly of Kupffer cells (KCs), the liver-resident macrophages and liver sinusoidal endothelial cells (LSECs). Whereas KCs facilitate the removal of larger blood-borne particles by phagocytosis, LSECs provide clearance of small particles (< 200 μm) and macromolecules by receptor-mediated endocytosis [23]. Both these cell types are central to the liver’s barrier function and its ability to clear the blood from gut-derived pathogens and dietary products, but also from a multitude of circulating degradation products, damaged cells and toxins [23]. Although many of the cleared compounds are potential inducers of inflammation [19], their removal by liver cells is typically not associated with inflammation, and inflammatory immune responses are actually often actively suppressed [24]. Indeed, liver tolerance was first demonstrated by showing that immune responses to orally ingested antigens are specifically suppressed in the liver, as the observed oral tolerance was abrogated when portal blood flow was diverted from the liver by portosystemic shunt [25]. Moreover, it was shown that skin allografts, which normally are rapidly rejected, were accepted when co-transplanted with allogeneic liver from the same donor [26]. Finally, it was shown that ectopic expression of a myelin autoantigen in the liver, which was facilitated by gene transfer to hepatocytes, provided protection from autoimmune neuroinflammatory disease [27]. These findings demonstrated that the liver features a profound capacity to induce immune tolerance, which is mainly established through suppressive effects on T cells and the ability to induce regulatory T cells that will be further explained below in the context of the respective liver cell types. Presumably, liver tolerance is a necessary adaptation to its constant exposure to numerous dietary and microbial derived antigens. Indeed, one might speculate that if the liver were more inclined towards inflammation, as other organs are, it would probably constantly be inflamed [24].

Liver tolerance is owed to a unique microenvironment rich in tolerogenic antigen-presenting cells and regulatory mediators [28, 29]. Moreover, the hepatic anatomy determines a relatively slow blood flow in the hepatic sinusoids that facilitates interactions between liver antigen-presenting cells and passing lymphocytes, which typically result in the induction of tolerance [28, 29]. Nonetheless, hepatic tolerance can be broken and inflammation be induced upon sensing of liver infections [24, 30, 31]. The hepatic microenvironment is established through mutual interactions between the resident cell types that determine cellular identity and zonation-specific phenotype [32, 33]. The tolerogenic microenvironment of the liver is composed of several types of antigen-presenting cells, which might qualify for being harnessed for antigen-specific immunotherapy. In the following, we briefly sum up the tolerance-inducing functions of the different liver cell types; for more detailed description, we refer to recent reviews on this subject, such as those in [24, 28, 29].

### Hepatocytes

Hepatocytes are the parenchymal cells of the liver performing the multiple metabolic tasks of the liver. However, hepatocytes can also interact with T cells and function as antigen-presenting cells [34]. Although MHC I expression is low, hepatocytes can prime CD8 T cells, which mostly results in CD8 T cell death [35, 36] or profound unresponsiveness even in infection [37]. MHC II expression is low or absent in steady-state [38], but can be upregulated in inflammatory conditions, rendering hepatocytes functional antigen-presenting cells that promote tolerance rather than inflammation [39, 40]. Besides direct antigen presentation by hepatocytes, it is conceivable that hepatocytes can also deliver antigen to neighbouring professional antigen-presenting cells, e.g. by trogocytosis or extracellular vesicles [41–43]. Indeed, it has been shown that tolerance to hepatocellular antigens can be lost following depletion of hepatic macrophages [44].

### Kupffer cells (KCs)

KCs are the liver-resident macrophages, which represent more than 80% of all tissue-resident macrophages in humans [24], and account for about 20% of the non-parenchymal liver cells [29]. KCs reside in the lumen of the hepatic sinusoids (Fig. 2) where they can interact with passing lymphocytes and function as antigen-presenting cells. However, compared to conventional antigen-presenting cells, KCs express only low levels of MHC II and co-stimulatory molecules, and produce anti-inflammatory prostaglandins, hence inducing anti-inflammatory and tolerogenic activation of T cells [45]. Accordingly, it was demonstrated that antigen-delivery to KCs with microparticles could induce antigen-specific tolerance and protection from antigen-driven kidney inflammation [46]. Importantly, however, in a state of liver inflammation and injury, microparticle-mediated antigen-delivery to KCs was no longer tolerogenic, but pro-inflammatory, due to plasticity and inflammatory activation of KCs and of monocyte-derived macrophages that were recruited to the inflamed liver [46]. Moreover, a KC subset was recently shown to revert hepatic CD8 T cell tolerance upon sensing of IL-2 [47]. Thus, although KCs are potent tolerance inducers in homoeostatic conditions, they are not ideal mediators of antigen-specific immunotherapy, due to their plasticity in aberrant conditions (Fig. 1).

### Liver dendritic cells (DCs)

The liver hosts all major DC subtypes, both of the conventional (cDC) and the plasmacytoid (pDC) lineages, of which the pDC population is over-represented as compared to secondary lymphoid organs [28, 29]. Liver DCs reside mainly in the portal field and the perivenous space, along with few DCs scattered throughout the parenchyma [28, 29]. Thus, liver DCs primarily act as sentinels in the tissue (Fig. 2). As these sentinels are not in direct contact with the hepatic blood flow, they can only interact with liver-infiltrating, not with circulating lymphocytes. In homoeostatic conditions, liver DCs are predominantly immature cells that promote immune tolerance, partly by producing IL-10 [28, 48, 49]. However, upon perception of infection or cell damage through conserved pattern recognition receptors, liver DCs become activated and in that state promote inflammatory T cell responses [24, 50]. Thus, liver DCs display high plasticity in perturbed tissue conditions, limiting their usefulness as mediators of antigen-specific immunotherapy (Fig. 1).

### Liver sinusoidal endothelial cells (LSECs)

LSECs line the hepatic sinusoids, but, unlike vascular endothelial cells, do not have a basal membrane, and feature fenestrations facilitating substance exchange between blood and the hepatocytes beneath the endothelium (Fig. 2). Similar to KCs, LSECs express low levels of MHC II and co-stimulatory molecules, but high levels of co-inhibitory molecules such as programmed death ligand-1 [51, 52]. Accordingly, stimulation of CD4 T cells by LSECs typically results in the suppression of inflammatory activities [53, 54]. Moreover, LSECs have been even found to induce a suppressive phenotype in conventional CD4 T cells [55]. Furthermore, owed to their ability to tether TGFb to their outer cell membrane, LSECs can effectively induce Foxp3-expressing regulatory T cells that have profound immunosuppressive capacity [14]. Accordingly, it has been demonstrated that nanoparticle-mediated delivery of myelin peptides to LSECs induced antigen-specific immune suppression and provided protection from myelin-driven CD4 T cell–mediated neuroinflammation [56].

LSECs are also highly effective inducers of CD8 T cell tolerance [52, 57], owed to their remarkable capacity to cross-present antigens taken up by endocytosis [57, 58]. LSECs are also able to induce CD8 memory T cells, which remain non-responsive during steady-state and require strong signals for re-activation [59, 60]. Accordingly, nanoparticle-mediated delivery of MHC I–restricted autoantigen peptide was found to induce antigen-specific CD8 T cell tolerance and protection from antigen-driven cholangitis [61]. Importantly, although it was found that LSECs acquire enhanced immunogenicity in liver fibrosis [62], it was also shown that tolerance induction by LSECs was robust when stimulated with inflammation inducers and overcome only by viral infection [31]. Thus, in contrast to other professional antigen-presenting cells, including those in the liver, LSECs display a low plasticity and a high resilience towards external inflammatory stimuli. Therefore, LSECs qualify well as mediators of tolerance induction in vivo; hence, antigen-delivery to LSECs is a promising strategy for antigen-specific immunotherapy (Fig. 1).

## Antigen-specific immunotherapies harnessing hepatic tolerance

Several methods have been designed that utilise liver tolerance for the development of antigen-specific immunotherapies. Current approaches can be classified into the categories ‘gene therapy’, ‘antigen-loaded erythrocytes’ and ‘antigen-loaded particles’, as elaborated below. Table 1 lists some approaches currently followed for translation into human therapies.

**Table 1:** Some current approaches to antigen-specific immunotherapy for autoimmune diseases harnessing liver tolerance

Company	Ag-conjugation	Liver target cell	Clinical trial
Anokion	Erythrocytes	KC	KAN-101 completed (celiac disease) ANK-700 (multiple sclerosis)
Cellerys	Erythrocytes	KC	
Cour	PLGA-NP	KC	CNP-101 (celiac disease) CNP-104 (primary biliary cholangitis)
Dendright	Liposomes	DC	
Selecta	PLG-NP	KC/LSEC/DC	
Topas	PMAOD-NP	LSEC	TPV11 (pemphigus vulgaris)

### Gene therapy

Liver-targeted gene therapy has been classically explored for several years as a treatment for genetic disorders, such as haemophilia A and B [63]. Typically, adeno-associated virus (AAV) is used as a vector to transfer genes to hepatocytes that then compensate for a genetic mutation underlying the inherited disorder [64, 65]. Importantly, AAV-based therapies have already been approved in Europe and the USA, increasing the prospects of regulatory approval also for AAV-based antigen-specific immunotherapies [65]. However, immunogenicity of vector and transgene are still a limitation of this method, providing an obstacle to long-term transgene expression in patients [64, 66]. In mouse models, however, immunogenicity could be avoided, when expression of the transferred gene was restricted to hepatocytes [67–70], once more illustrating the potential of liver tolerance.

Using a mouse model for multiple sclerosis [71], a seminal study demonstrated that gene transfer to hepatocytes can provide effective antigen-specific immunotherapy to treat autoimmune disease [27]. Gene transfer of myelin antigen to hepatocytes, which was either achieved by microinjection of a transgene construct into fertilized mouse eggs, or by adenoviral or hydrodynamics-based gene transfer in adult mice, resulted in the generation of myelin antigen-specific regulatory T cells and protection from autoimmune neuroinflammation [27]. Subsequently, gene transfer of the insulin B (9–23) antigen to hepatocytes was successfully applied to treat nonobese diabetic (NOD) mice [72], which spontaneously develop type 1 diabetes [73]. Using a liver-specific promotor, lentiviral gene transfer was used to transiently express InsB_9-23_ specifically in hepatocytes, inducing increased numbers of regulatory T cells, and decreased infiltration and destruction of pancreatic islets, thus retaining normal levels of insulin production and normoglycemia in treated mice [72]. Moreover, AAV-mediated hepatic gene transfer of myelin oligodendrocyte glycoprotein could also induce induction of regulatory T cells and prevent experimental neuroinflammation [74]. Treatment of mild symptoms was also possible, whereas the treatment of severe symptoms required additional immunosuppression [74].

Thus, antigen-specific immunotherapy based on gene transfer to hepatocytes is a promising approach for the treatment of autoimmune diseases. However, its clinical application is currently limited due to immunogenicity of vector and transferred gene. A possible solution to these problems might be found in the emerging development of nanoparticles as vectors for gene transfer (see below) [75].

### Antigen-loaded erythrocytes

Aging erythrocytes undergo a programmed form of cell death, called eryptosis [76], during which they translocate phosphatidylserine to their outer membrane, facilitating their phagocytosis by macrophages [77]. As we have argued above, Kupffer cells of the liver represent more than 80% of all tissue macrophage in humans. Therefore, the liver is a major site of erythrocyte phagocytosis, and erythrocytes might thus be a suitable vector to deliver autoantigens to Kupffer cells for the purpose of antigen-specific immunotherapy. This approach was explored by Kontos et al., who conjugated peptides or whole proteins to erythrocytes, resulting in deletion of antigen-specific T cells and tolerance in a diabetes model [78]. Deletional tolerance seemed to depend on the PD-L1 molecule [79], which is constitutively expressed on Kupffer cells [80]. Importantly, this approach to liver tolerance has also been used to prevent the generation of anti-drug antibodies [81], which are a major cause for treatment failure of biological therapeutics [82].

Thus, antigen-specific immunotherapy based on antigen-coupling to erythrocytes is a promising approach for the treatment of autoimmune diseases. However, as it mostly relies on Kupffer cells and other macrophages, which can display a high degree of plasticity under inflammatory conditions, there might be a risk of disease exacerbation in some patients. Currently, we do not have enough clinical data to really assess that risk, if there is one at all. Clinical trials are needed to further explore this promising approach.

### Antigen-loaded nanoparticles

Nanomedicine is a rapidly emerging field utilising nanoscale materials for pharmaceutical or diagnostic purposes, and several approaches have been taken to use nanomaterials for antigen-specific immunotherapy of autoimmune diseases [83]. Their physico-chemical properties make nanoparticles ideal vectors for tolerance-inducing delivery of autoantigen peptides. Compared to erythrocytes, nanoparticles have a much larger surface-to-volume ratio, providing a greatly increased capacity to carry peptide cargo. Moreover, nanoparticles can be designed to facilitate targeted delivery to specific cell types [84, 85] or co-delivery of tolerance-inducing mediators [83].

Latex microparticles have been used to selectively target antigen peptides to Kupffer cells, resulting in expansion of regulatory T cells and antigen-specific disease attenuation in a model of autoimmune kidney damage [46]. However, when subjected to inflammatory, liver-damaging treatments with either CCl_4_ or a methionine-choline-deficient diet, tolerance-induction was abrogated, mainly owed to the plasticity and inflammatory activation of the hepatic phagocytes [46]. This study is of particular relevance, as various other methods for tolerance induction use micro- or nanocarriers that are preferentially phagocytosed by macrophages [83]. Examples of these macrophage-targeting nanomaterials are polystyrene or poly(lactide-*co*-glycolide), which have been demonstrated to induce regulatory T cells and effective antigen-specific protection from autoimmune neuroinflammation [86] or type 1 diabetes [87]. Although not primarily designed to selectively target Kupffer cells, these nanomaterials strongly enrich in Kupffer cells, owed to their location and quantity. Therefore, it is of utmost importance to carefully select the patients to be considered for treatment with such nanotherapies, and closely monitor their safety. Nonetheless, even when applied to patients with healthy livers, a safety risk remains, as extrahepatic macrophages, which are likewise targeted by these nanomaterials, can also exhibit high plasticity [88]. Thus, these nanotherapies might be candidates for effective antigen-specific immunotherapy of autoimmune diseases, provided that they are safe.

Dendritic cell–targeting nanoparticles are also discussed as vectors for antigen-specific immunotherapy [89], but, like macrophages, dendritic cells can exhibit high plasticity. Thus, similar safety concerns as for macrophages apply to dendritic cells. In any case, liver dendritic cells are difficult targets for selective delivery with nanovectors, as these cells, as argued above, are not in direct contact with blood and are more secluded in the liver parenchyma, although their targeting by liposomes has been reported [90].

Amphiphilic polymer-coated nanocrystals have been found to be taken up with high selectivity by LSECs [56]. These LSEC-targeting nanoparticles have been reported to provide generation of regulatory T cells and antigen-specific protection from CD4 T cell–driven autoimmune neuroinflammation [56]. Moreover, owed to the ability of LSECs to cross-present peptides that were delivered with LSEC-targeting nanoparticles, this approach could also be used to induce antigen-specific CD8 T cell tolerance and protection from CD8 T cell–mediated cholangitis [61]. It is likely that also other types of nanoparticles, notably those that are small, can be taken up by LSECs [91], but thus far a high selectivity has not been reported. Given their relatively low plasticity, LSECs are very promising target cells for antigen-specific immunotherapies, as they offer both high efficacy and a good safety profile.

## Conclusions

The liver hosts several types of antigen-presenting cells that are effective inducers of immune tolerance. These inherently tolerogenic liver cell types can be harnessed by several methods to provide effective antigen-specific immunotherapy of autoimmune diseases, as has been demonstrated in various preclinical models. Personally, we currently prefer autoantigen delivery to hepatocytes or LSECs over delivery to KCs or DCs, as the latter exhibit considerably higher plasticity under non-homoeostatic conditions, raising potential safety issues. Thus, we find nanoparticles as vectors for gene transfer to hepatocytes, and nanoparticles as vectors for antigen peptide delivery to LSECs exciting and currently most promising. However, it is important to carry on exploring all these methods, as their efficacy and safety in humans will eventually only become evident in the course of their clinical translation.
